# Optimized Ship-Radiated Noise Feature Extraction Approaches Based on CEEMDAN and Slope Entropy

**DOI:** 10.3390/e24091265

**Published:** 2022-09-08

**Authors:** Yuxing Li, Bingzhao Tang, Shangbin Jiao

**Affiliations:** 1 School of Automation and Information Engineering, Xi’an University of Technology, Xi’an 710048, China; 2 Shaanxi Key Laboratory of Complex System Control and Intelligent Information Processing, Xi’an University of Technology, Xi’an 710048, China

**Keywords:** slope entropy, dispersion entropy, CEEMDAN, ship-radiated noise, feature extraction

## Abstract

Slope entropy (Slopen) has been demonstrated to be an excellent approach to extracting ship-radiated noise signals (S-NSs) features by analyzing the complexity of the signals; however, its recognition ability is limited because it extracts the features of undecomposed S-NSs. To solve this problem, in this study, we combined complete ensemble empirical mode decomposition with adaptive noise (CEEMDAN) to explore the differences of Slopen between the intrinsic mode components (IMFs) of the S-NSs and proposed a single-IMF optimized feature extraction approach. Aiming to further enhance its performance, the optimized combination of dual-IMFs was selected, and a dual-IMF optimized feature extraction approach was also proposed. We conducted three experiments to demonstrate the effectiveness of CEEMDAN, Slopen, and the proposed approaches. The experimental and comparative results revealed both of the proposed single- and dual-IMF optimized feature extraction approaches based on Slopen and CEEMDAN to be more effective than the original ship signal-based and IMF-based feature extraction approaches.

## 1. Introduction

With the increasing complexity of the marine environment, the classification and identification of underwater acoustic targets are of great importance in areas such as national defense and the exploitation of marine resources [1,2]. A ship-radiated noise signal (S-NS), as the focus of research in the field of underwater acoustics, contains a variety of information such as ship target type, tonnage, speed, and so on, which is helpful in the recognition, classification, and tracking of ship targets [3,4]. The key technology of S-NS classification is “feature extraction”, and further development of the feature extraction technology is conducive to improving the classification performance of S-NSs [5,6,7].

The feature extraction approaches of S-NSs are generally divided into two categories. The first category involves directly extracting the features of the undecomposed S-NSs. Such feature extraction approaches of S-NS usually use traditional features and entropy-based features. Traditional features mainly include frequency, energy, spectrum, and so on [8,9,10,11], while entropy-based features consist of dispersion entropy (DE) [12], reverse dispersion entropy (RDE) [13], and fluctuation dispersion entropy (FDE) [14]. Slope entropy (Slopen) was proposed to analyze the complexity of the signal in 2019 [15], and first applied to underwater acoustics [16]. In [16], the S-NS feature extraction approach was proposed based on Slopen; the performance revealed that Slopen possessed the highest classification performance compared to DE, RDE, and FDE. From the above, we can realize the following: (i) compared with some traditional features, entropy-based feature extraction approaches are superior, and (ii) compared with other entropies, Slopen as a new complexity feature can better distinguish S-NSs.

The second category is to extract the features of mode components, which are obtained with a decomposition algorithm [17,18]. Currently, there are many decomposition algorithms, such as empirical mode decomposition (EMD) [19], ensemble empirical mode decomposition (EEMD) [20], complete ensemble empirical mode decomposition with adaptive noise (CEEMDAN) [21], and variational mode decomposition (VMD) [22]. Similar to the first category of approaches, these features are classified as traditional features or entropy-based features. In 2016, Li et al., used EMD to process S-NSs and combined it with permutation entropy (PE) [23] to identify different S-NSs [24]. Other researchers, in [25,26], replaced EMD with EEMD and proposed the feature extraction approaches of S-NS using sample entropy and multi-scale PE, respectively; their results show that the S-NS feature extraction approach based on EEMD had a higher recognition rate. To improve the classification performance, [27] employed CEEMDAN and energy entropy to classify the S-NSs, and the results indicated that the feature extraction approach based on CEEMDAN can accurately recognize S-NSs. In addition, Yang et al., presented a novel S-NS feature extraction approach based on VMD and FDE [28], and the experimental results showed that the feature extraction approach based on VMD is better than that based on EMD and EEMD. In summary, we draw the following conclusions from the literature: (i) compared with the first category of feature extraction approaches, mode components-based feature extraction approaches have better separability and classification performance; and (ii) within the second category of feature extraction approaches, the entropy-based feature is better than other features—VMD and CEEMDAN have more advantages for S-NS feature extraction than EMD and EEMD. However, a limitation of VMD is that its influence parameters need to be set in advance [29,30].

The main contributions of this study are as follows: (i) CEEMDAN is an adaptive decomposition algorithm, which overcomes the parameter selection limitation of VMD; (ii) compared with other traditional features and entropy-based features, Slopen has better recognition performance in the first category of S-NS feature extraction approaches; and (iii) this study used the advantages of CEEMDAN and Slopen to propose optimized S-NS feature extraction approaches for single-IMF and dual-IMFs. The rest of this paper is structured as follows: Section 2 introduces the concepts of CEEMDAN and Slopen; Section 3 describes the main steps of the proposed single- and dual-IMF optimized feature extraction approaches; Section 4 presents the experimental validations using three comparative experiments; and finally, Section 5 provides the conclusion.

## 2. Theoretical Background

### 2.1. CEEMDAN

There is some white noise in the mode components that is obtained by the decomposing signal with EEMD and complementary ensemble empirical mode decomposition (CEEMD), which impacts signal analysis and processing. Therefore, in order to solve this problem, CEEMDAN was proposed.

CEEMDAN changes the way that noise is added. The algorithm adds the intrinsic mode components (IMFs) with auxiliary noise after EMD decomposition to the original signal, but does not directly add Gaussian white noise. Additionally, CEEMDAN changes the method of determining the IMF. CEEMDAN determines a mode component every time noise is added, which is an iterative process, rather than decomposing the noisy signal only once to obtain the IMF of each order. The specific steps are as follows:

**Step 1:** Add Gaussian white noise $\varepsilon_{0}*V^{j}\left(t\right)$ to the initial signal *x(t)* to obtain a new signal *y(t)* and apply the EMD algorithm to decompose the signal *y(t)* to obtain the first mode component of CEEMDAN:
  (1) $$C_{1}(t)=\frac{1}{N}*\sum_{j=1}^{N}C_{1}^{j}\left(t\right),j=1,2,\ldots N$$
  where $\varepsilon_{0}$ is the standard deviation of the added white noise, $V^{j}$ is Gaussian white noise with unit variance under the condition of the *j*-th ensemble number, *N* is the total ensemble, and $C_{1}^{j}$ is defined as the *j*-th mode component of EMD decomposition.
**Step 2:** Determine the residual component$\;r_{1}\left(t\right)$ after the first decomposition.
  (2) $$r_{1}\left(t\right)=x\left(t\right)-C_{1}(t)$$
**Step 3:** Use the residual signal after adding white noise as a new signal $r_{1}\left(t\right)+\varepsilon_{0}*E_{1}(V^{j}\left(t\right)$) to be decomposed, and, through the same process, obtain the second mode component $C_{2}$(t) and residual component $r_{2}\left(t\right)$.
  (3) $$C_{2}\left(t)=\frac{1}{N}*\sum_{j=1}^{N}E_{1}\left(r_{1}\left(t\right)+\varepsilon_{0}*E_{1}(V^{j}\left(t\right)\right)\right)$$
  (4) $$r_{2}\left(t\right)=r_{1}\left(t\right)-C_{2}\left(t\right)$$
  where $E_{j}$(*) is the function of extracting the *j*-th IMF, which is decomposed of the EMD decomposition.
**Step 4:** Repeat the above steps until the stop condition is met. That is, once the residual signal is a monotonic function the cycle ends to obtain the *K* IMF components and remaining residual components $r\left(t\right)$. The original signal $x\left(t\right)$ is decomposed as follows:
  (5) $$x\left(t\right)=\overset{K}{\underset{k=1}{\sum }}C_{k}\left(t\right)+r\left(t\right)$$

### 2.2. Slopen

**Figure 1:**
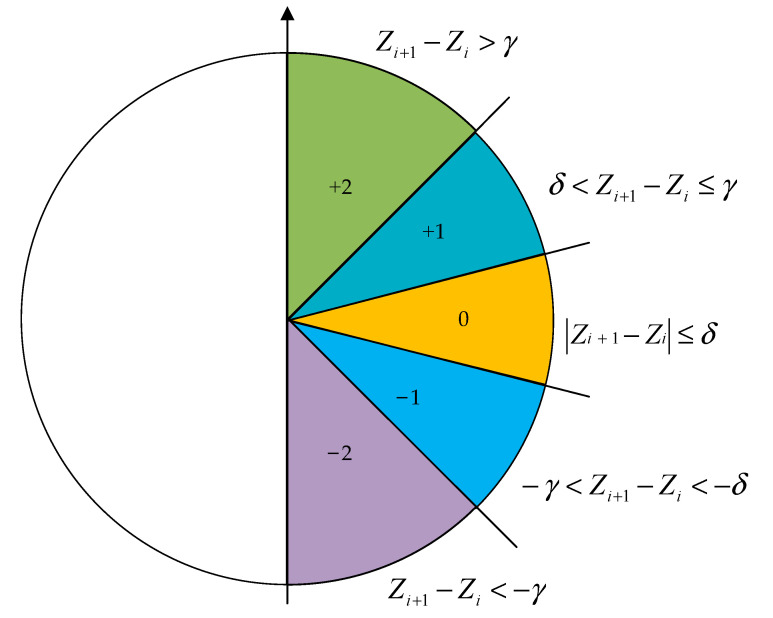
The division of symbol patterns.

Slopen is an algorithm that can characterize the complexity of a time series. It is primarily based on single-threshold and symbolic patterns, where every symbol is largely determined by the distinction between consecutive samples of the input time series [31,32]. The specific steps of the slope entropy algorithm are as follows:

**Step 1:** Given a time series $Z=\left\{z_{i},\;i=1,\;2,\;\ldots ,\;n\right\}$, the extracted sequences are $Z_{1}=\left\{z_{1},\;z_{2},\;\ldots ,z_{m}\right\},\;Z_{2}=\left\{z_{2},\;z_{3},\;\ldots ,\;z_{m+1}\right\}$, …, $Z_{k}=\left\{z_{k},\;z_{k+1},\;z_{n}\right\}$, where the embedded dimension is m and k=n−m+1.
**Step 2:** Dividing symbolic patterns by two thresholds (γ and δ). If $z_{i+1}-z_{i}>\delta$, the symbol is defined as +2; if $\gamma <z_{i+1}-z_{i}<\delta$, the symbol is defined as +1; if $\left|z_{i+1}-z_{i}\right|\leq \gamma$, the symbol is defined as 0; if $-\delta <z_{i+1}-z_{i}<-\gamma$, the symbol is defined as −1; and if $z_{i+1}-z_{i}<-\delta$, the symbol is defined as −2, where δ>γ>0. Figure 1 shows the division of symbol patterns.
**Step 3:** The symbol pattern sequences obtained from the previous step are $Y_{1}=\left\{y_{1},\;y_{2},\;\ldots ,\;y_{m-1}\right\}$, $Y_{2}=\left\{y_{2},\;y_{3},\;\ldots ,\;y_{m}\right\}$, …, $Y_{k}=\left\{y_{k},\;y_{k+1},\;\ldots ,\;y_{n-1}\right\}$, where $y_{j}$ is the symbol corresponding to $z_{j+1}-z_{j}$ and k=n−m+1.
**Step 4:** The total number of types of symbol pattern sequences is recorded as $J=5^{m-1}$, the corresponding number of different types of sequences is recorded as $t_{i},\;i=1,\;2,\;\ldots ,\;$*S*, and the relative frequency of occurrence is recorded as$\;p_{i}$:
  (6) $$p_{i}=\frac{t_{i}}{J},\;i=1,\;2,\;\ldots ,\;S$$
**Step 5:** Therefore, Slopen is defined as follows:
  (7) $$H_{s}\left(m\right)=-\overset{S}{\underset{i=1}{\sum }}p_{i}lnp_{i}$$
  where $p_{i}$ is the relative frequency of occurrence.

## 3. Feature Extraction Approach

**Figure 2:**
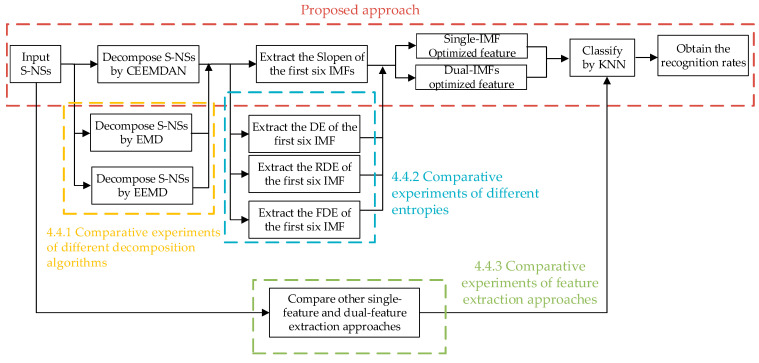
The flow chart of feature extraction for S-NSs.

Based on the theoretical analyses of CEEMDAN and Slopen, the signal-IMF and dual-IMF optimized feature extraction approaches for S-NSs, termed “CEEMDAN-Single-Slopen” and “CEEMDAN-Dual-Slopen,” were proposed, respectively. The flow chart depicting the feature extraction for S-NSs is shown in Figure 2 and the specific steps of the study were as follows:

(1) Four types of S-NSs were obtained and used as the study subjects for the experiments;
(2) S-NSs were decomposed into several IMFs using CEEMDAN; subsequently, using Slopen as the feature, the first six IMFs were selected for feature extraction; in addition, comparisons with some classical decomposition algorithms, such as EMD and EEMD, were made;
(3) The Slopens of the first six IMFs were extracted and the single-IMF and dual-IMF optimized feature extraction experiments were carried out; concurrently, the DE, RDE, and FDE of the IMF were extracted, respectively, and comparative experiments were performed.
(4) KNN was adopted to classify four types of S-NSs; then, the recognition rates were obtained and compared with the other single-feature and dual-feature extraction approaches based on the original S-NSs.

## 4. Feature Extraction and Classification of S-NSs

### 4.1. Four Types of S-NSs

**Figure 3:**
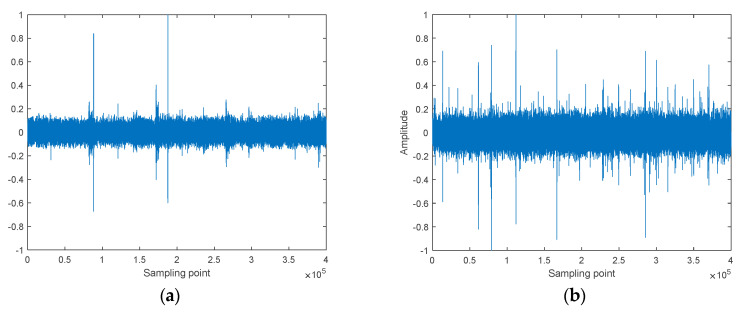

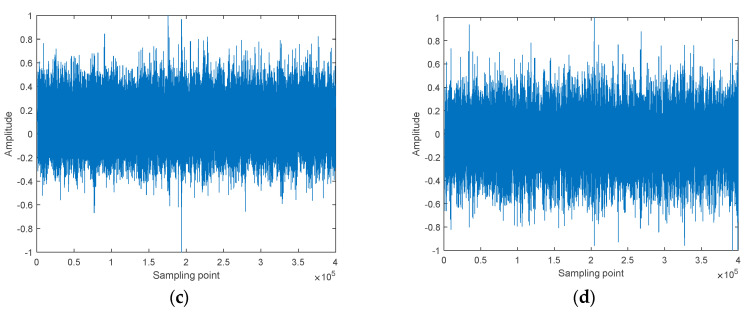
The normalized waveform of the four types of S-NSs. (**a**) Ship−1; (**b**) Ship−2; (**c**) Ship−3; (**d**) Ship−4.

Four types of S-NSs were used in the feature extraction experiments, consisting of Ship-1, Ship-2, Ship-3, and Ship-4. Ship-1 and Ship-2 were obtained from a database named ShipsEar (Available at: http://atlanttic.uvigo.es/underwaternoise/, accessed on 13 July 2022) and represent an ocean liner and a motorboat, respectively. Ship-3 and Ship-4 were obtained from the official website of the National Park Service (Available at: https://www.nps.gov/glba/learn/nature/soundclips.htm, accessed on 15 July 2022) and represent an Alaska state ferry and a cruise ship, respectively. For all four types of S-NSs, the sampling point’s length was 400,000. Figure 3 shows the normalized waveform of the four types of S-NSs.

### 4.2. The Decomposition of S-NSs

**Figure 4:**
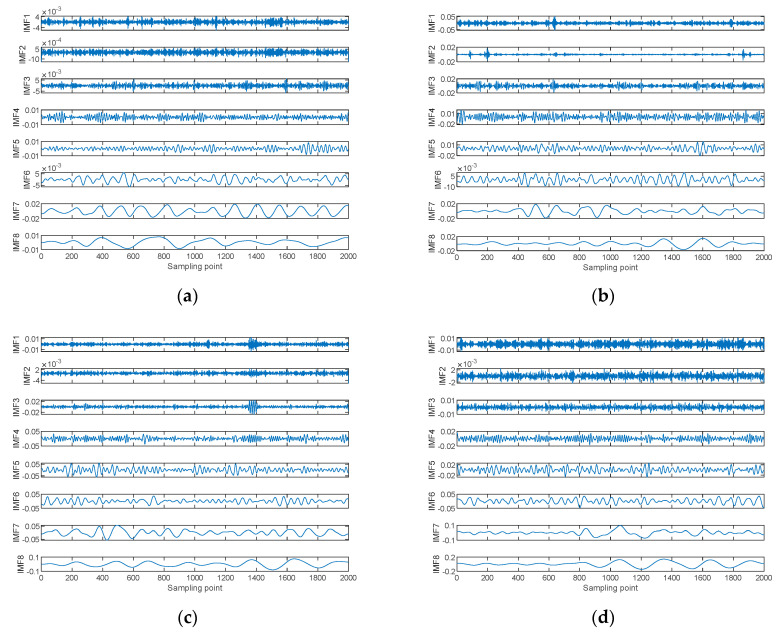
The decomposition results for one sample of the four types of S-NSs decomposed by CEEMDAN. (**a**) Ship−1; (**b**) Ship−2; (**c**) Ship−3; (**d**) Ship−4.

All four types of S-NS were decomposed into several IMFs using CEEMDAN. For each type of S-NS, there were 200 samples and each sample consisted of 2000 sampling points. For CEEMDAN, the noise standard deviation was set to 0.2, the number of noise additions was 500, and the maximum number of sifting iterations permitted was 3000. The decomposition results for one sample of the four types of S-NSs decomposed by CEEMDAN are shown in Figure 4.

### 4.3. Feature Extraction

**Figure 5:**
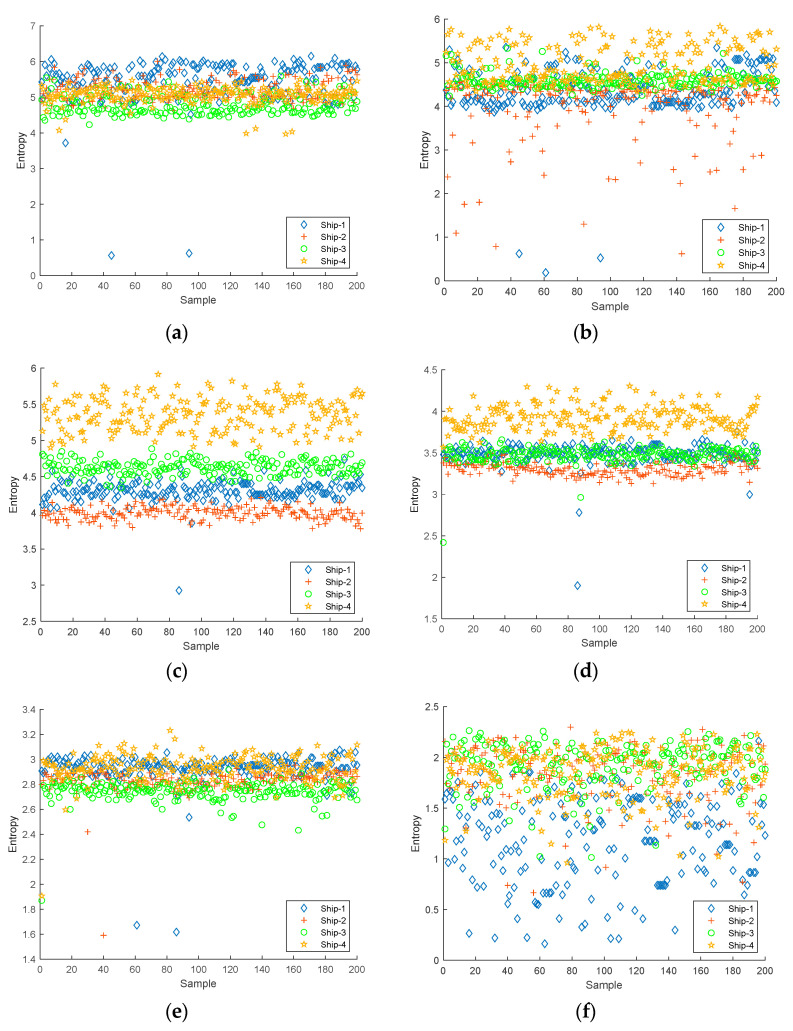
The feature distributions of Slopen in each IMF of four types of S-NSs. (**a**) IMF1; (**b**) IMF2; (**c**) IMF3; (**d**) IMF4; (**e**) IMF5; (**f**) IMF6.

**Figure 6:**
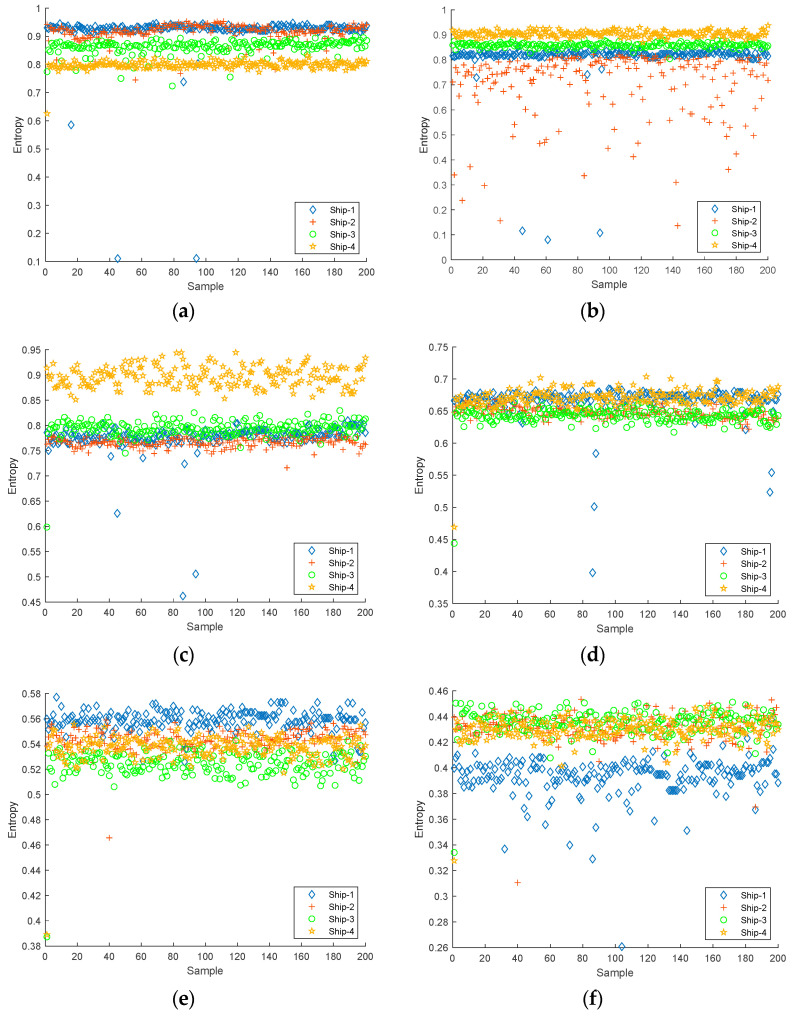
The feature distributions of DE in each IMF of four types of S-NSs. (**a**) IMF1; (**b**) IMF2; (**c**) IMF3; (**d**) IMF4; (**e**) IMF5; (**f**) IMF6.

**Figure 7:**
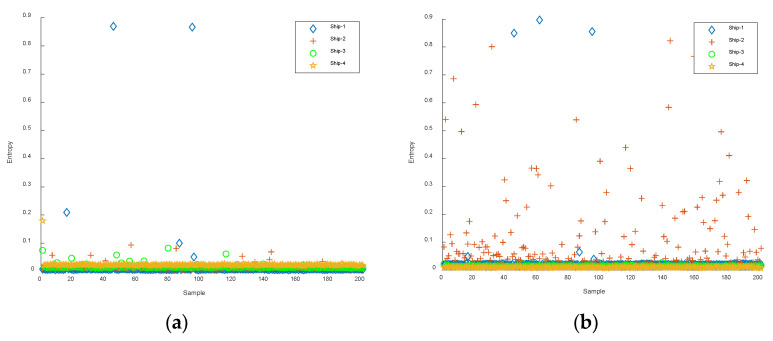

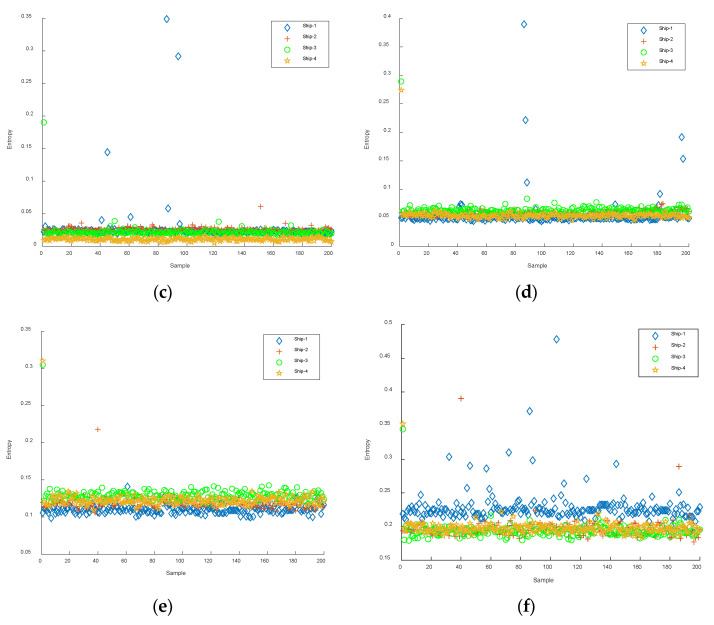
The feature distributions of RDE in each IMF of four types of S-NSs. (**a**) IMF1; (**b**) IMF2; (**c**) IMF3; (**d**) IMF4; (**e**) IMF5; (**f**) IMF6.

**Figure 8:**
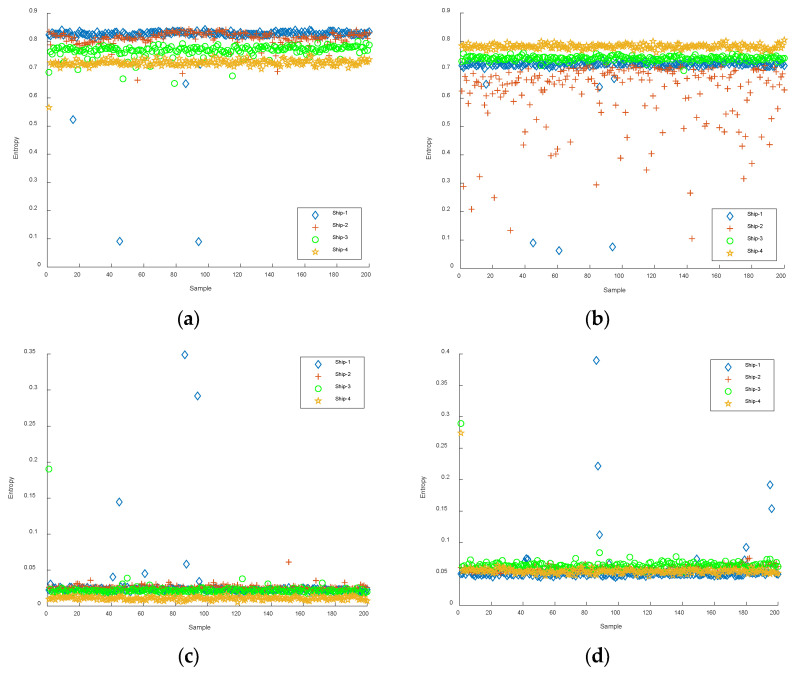

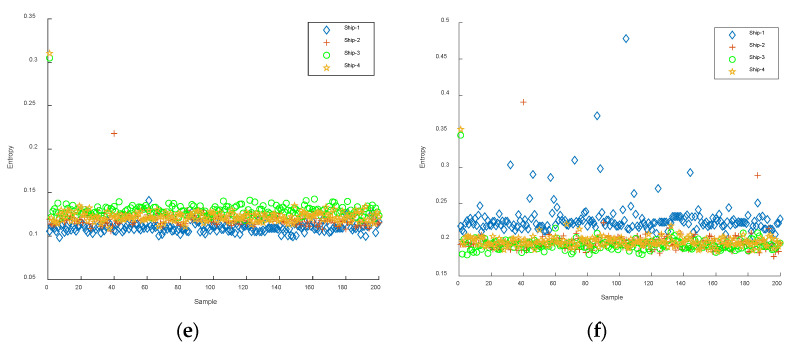
The feature distributions of FDE in each IMF of four types of S-NSs. (**a**) IMF1; (**b**) IMF2; (**c**) IMF3; (**d**) IMF4; (**e**) IMF5; (**f**) IMF6.

The first six IMFs obtained from CEEMDAN were used as the object of the experiment for feature extraction. The Slopen of each IMF was extracted separately, and the DE, RDE, and FDE of each IMF were extracted for comparison. The feature distributions of Slopen, DE, RDE, and FDE in every IMF of four types of S-NSs are presented in Figure 5, Figure 6, Figure 7 and Figure 8.

As can be seen from Figure 5, Figure 6, Figure 7 and Figure 8, within the four types of entropies, the distributions of SN-Ss in different IMFs were relatively chaotic and the overall entropy value became smaller from IMF1 to IMF6. Compared with the other five IMFs of Slopen, the Slopen values of the four types of S-NS in IMF3 had more apparent differences and less overlap in their distributions. For the same IMF, the Slopen value was higher compared to DE, FDE, and RDE. For DE, compared to IMF3 to IMF6, the entropy values of Ship-3 and Ship-4 in IMF1 and IMF2 were significantly different. The difference between the DE values of Ship-2 and Ship-3 was small from IMF3 to IMF6. The RDE values of the four types of S-NSs from IMF1 to IMF6 were very similar compared to the other three types of entropies, and the RDE value of the majority of samples was below 0.25. Regarding FDE, the entropy values of the SN-Ss in IMF1, IMF2, IMF3, and IMF4 were very close to each other. Moreover, the entropy values of the first four IMFs were mostly within the range of 0.4–0.9, while the last two IMFs fluctuated between 0.08 and 0.36. Among the six IMFs, the differences between the distributions of the four types of S-NSs were most evident in IMF2.

### 4.4. Classification and Recognition

In this section, three experiments were conducted to confirm the effectiveness of the proposed approaches. In addition, the following sets of comparative experiments were conducted: (1) comparison experiments of mode decomposition with classical decomposition algorithms (including EMD and EEMD); (2) comparison experiments of entropy indicators with classical entropies (including DE, RDE, and FDE); and (3) comparison experiments of feature extraction approaches with feature extraction approaches (including single- and dual-feature extractions based on the original signal).

#### 4.4.1. Comparative Experiments of Different Decomposition Algorithms

To investigate the effect of different decomposition algorithms for single-IMF feature extraction, in addition to CEEMDAN, we also decomposed the S-NSs using EMD and EEMD, obtained the first six IMFs, extracted the Slopen from each IMF, and selected the optimized IMF. We labeled the comparative approaches as “EMD-Single-Slopen” and “EEMD-Single-Slopen”, respectively. There were 200 samples of each type of S-NS for KNN [33], of which the first 50 samples were used as training samples and the others as test samples. The average recognition rates of each IMF for three feature extraction approaches are shown in Table 1.

As shown in Table 1, under the single-IMF feature, compared with EMD-Single-Slopen and EEMD-Single-Slopen approaches, the CEEMDAN-Single-Slopen approach had the highest average recognition rate of 90.5%. Moreover, from IMF3 to IMF6, the average recognition rate of the CEEMDAN-Single-Slopen approach was higher than that of the two comparative feature extraction approaches. The highest average recognitions of EMD-Single-Slopen and EEMD-Single-Slopen were 60.3% and 50.8%, respectively, which were 30.2% and 39.7% lower than that of the CEEMDAN-Single-Slopen approach.

Since the recognition rates of the single-IMF optimized feature extraction approach were not high, experiments based on the dual-IMF optimized feature extraction were carried out. Similar to the single-IMF feature extraction experiments, and in contrast to CEEMDAN, we used EMD and EEMD to decompose the S-NSs. We then selected the first six IMFs, extracted the Slopen of any two IMFs, and selected the optimized combinations of dual-IMFs, which we named “EMD-Dual-Slopen” and “EEMD-Dual-Slopen”. For any dual-IMF extraction method, there were a total of 15 combinations for any two IMFs. The highest average recognition results of the dual-IMF optimized feature extraction approaches are shown in Table 2.

It can be found in Table 2 that under the dual-IMF feature, for different approaches, the combinations of IMFs were different. For example, for the CEEMDAN-Dual-Slopen approach, the highest recognition rate was obtained by extracting IMF3 and IMF5; the highest average recognition rate based on CEEMDAN-Dual-Slopen was 97.6%, which was 12.1%, and 29.3% higher than that of the EMD-Dual-Slopen and EEMD-Dual-Slopen approaches, respectively. Thus, the proposed CEEMDAN-Dual-Slopen approach is better than the other two dual-IMFs optimized feature extraction approaches. The experimental results reveal that in both the case of single-IMF or dual-IMF, the recognition rate of the proposed extraction approach based on CEEMDAN is better than that of EMD and EEMD.

#### 4.4.2. Comparative Experiments of Different Entropies

To explore the influence of different entropies in single-IMF feature extraction, while extracting the Slopen of each IMF, the DE, RDE, and FDE of each IMF were extracted separately for comparative analysis. The average recognition rate of every IMF of the four types of entropies are shown in Table 3.

**Figure 9:**
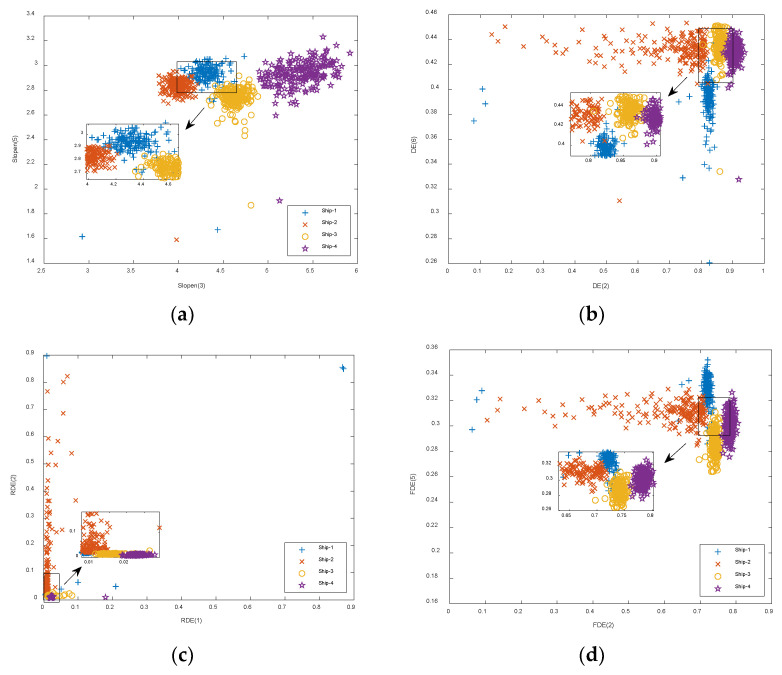
The feature distribution of the highest recognition rate of four types of entropies using the dual-IMF feature. (**a**) Slopen; (**b**) DE; (**c**) RDE; (**d**) FDE.

It can be seen from Table 3 that compared with the other three entropies, the IMF3 of Slopen had the highest recognition rate of 90.5%, which was 1%, 4.8%, and 0.2% higher than that of DE, RDE, and FDE, respectively. Overall, the recognition rates of the four types of entropies for each IMF were not high. To further improve the recognition rate, the dual-IMF extraction method was used to extract S-NSs. The feature distributions of the highest recognition rate of the four types of entropies under the dual-IMFs feature are presented in Figure 9, where Slopen(*i*) denotes the Slopen of the *i*-th IMF, and the same for DE, RDE, and FDE.

Figure 9 shows that, for Slopen, the distribution of features belonging to each type of S-NS was highly concentrated and there was little overlap. In addition, for DE, RDE, and FDE, the feature distribution of Ship-2 was highly scattered, and the entropy values of Ship-3 and Ship-4 were similar; especially for RDE, in which the entropy values for all four types of S-NSs were very close. The highest average recognition rates of the dual-IMFs of the four types of entropies are shown in Table 4.

From Table 4, it is clear that for the four types of entropy, all of the highest recognition rates were higher than 92%. The highest average recognition rate of the Slopen was the highest at 2%, 5.1%, and 2.8% higher than that of DE, RDE, and FDE, respectively. As such, the proposed CEEMDAN-Dual-Slopen approach is significantly better than the optimized feature extraction approaches based on the other three types of entropies.

#### 4.4.3. Comparative Experiments of Feature Extraction Approaches

Aiming to demonstrate the excellence of the proposed CEEMDAN-Single-Slopen approach, we compared four single-feature approaches which directly extracted the Slopen, DE, RDE, and FDE of the four types of S-NSs, respectively. The average recognition rates of the proposed CEEMDAN-Single-Slopen approach and the four single-feature extraction approaches are shown in Table 5.

As can be seen in Table 5, the CEEMDAN-Single-Slopen approach extracted the Slopen of IMF3, Slopen(3), which represented the highest average recognition rate among the six IMFs at 90.5%. The four single-feature approaches directly extracted the different entropies of S-NSs and had the highest average recognition rate of 78%, which is 12.5% lower than that of the CEEMDAN-Single-Slopen approach. Thus, the recognition rate of the CEEMDAN-Single-Slopen approach was much higher than the highest recognition rate of the four single-feature extraction approaches.

**Figure 10:**
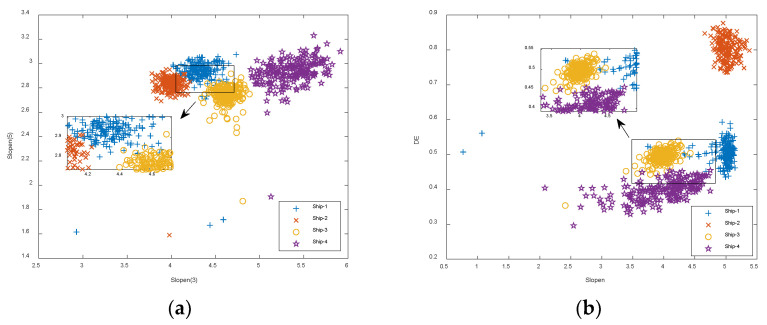

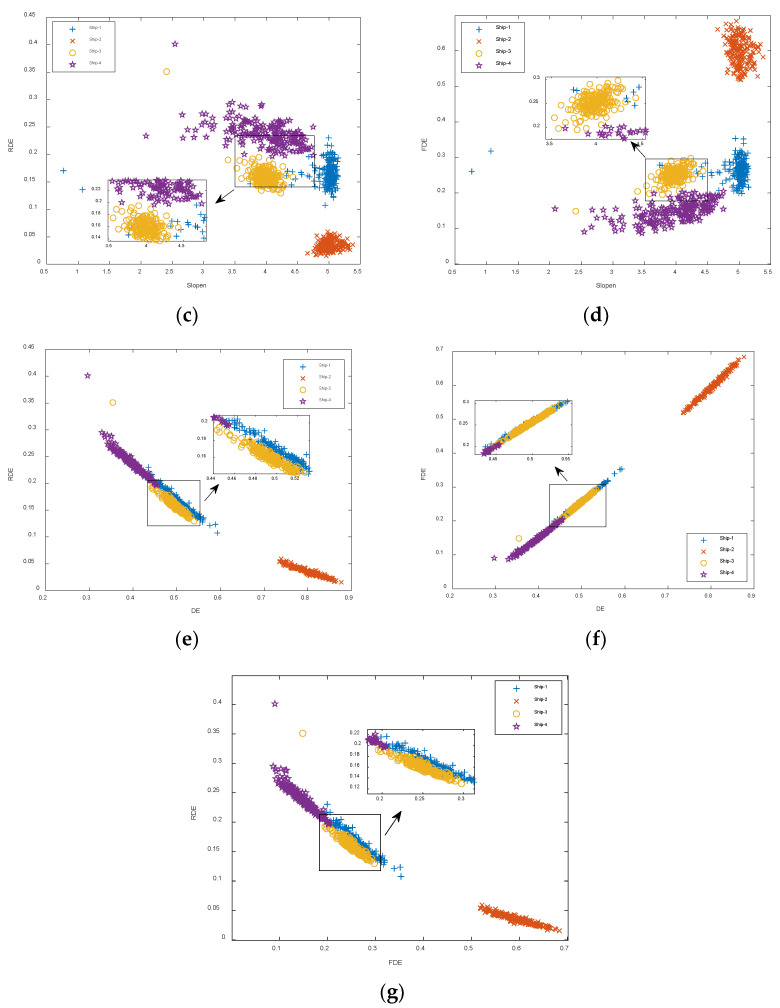
The distributions of the proposed CEEMDAN-Dual-Slopen approach and the dual-feature extraction approaches. (**a**) Slopen(3) and Slopen(5); (**b**) Slopen and DE; (**c**) Slopen and RDE; (**d**) Slopen and FDE; (**e**) DE and RDE; (**f**) DE and FDE; (**g**) FDE and RDE.

We also compared the dual-feature approaches, which directly extracted two of the entropies for the four types of S-NSs. The distributions of the proposed CEEMDAN-Dual-Slopen approach and the dual-feature extraction approaches are shown in Figure 10. In Figure 10a, Slopen(3) and Slopen(5) represent the Slopens of IMF3 and IMF5. In Figure 10b, Slopen and DE represent both the Slopen and DE of S-NSs, and so on for Slopen and DE, Slopen and RDE, etc.

As shown in Figure 10, for Slopen(3) and Slopen(5), Slopen and DE, and Slopen and RDE, compared to Slopen(3) and Slopen(5), the Ship-4 distribution was relatively more diffuse. For DE and RDE, DE and FDE, and FDE and RDE distributions, the distributions were mainly in the shape of bars. The distributions of Ship-1 and Ship-3 consistently overlapped each other in all seven feature approaches. The average recognition rates of the proposed CEEMDAN-Dual-Slopen approach and the dual-feature extraction approaches are shown in Table 6.

As can be seen from Table 6, in comparison to other dual-feature extraction approaches, the average recognition rate of the CEEMDAN-Dual-Slopen approach, based on Slopen(3) and Slopen(5), was the highest at 97.6%, which was 0.2%, 1.3%, 2.3%, 18.1%, 3.1%, and 5.1% higher than that of the dual-feature extraction approaches based on Slopen and DE, Slopen and RDE, Slopen and FDE, DE and RDE, DE and FDE, and RDE and FDE, respectively. To summarize the above findings, the proposed CEEMDAN-Single-Slopen and CEEMDAN-Dual-Slopen approaches are preferable to both the single-feature extraction approaches and the dual-feature extraction approaches based on S-NSs.

## 5. Conclusions

With the aim of improving the recognition of S-NSs, CEEMDAN decomposed the S-NSs into several IMFs. The Slopen was used as the feature of the IMFs, and two IMF-based feature extraction approaches for S-NSs were proposed. The experimental results of this study prove the effectiveness of the proposed approaches, and the main conclusions are as follows:

(1) Slopen was introduced as the new feature in the feature extraction of S-NSs; moreover, combined with CEEMDAN, this paper proposed CEEMDAN-Single-Slopen and CEEMDAN-Dual-Slopen approaches for S-NSs.
(2) Under the condition of a single feature, whether altering the decomposition algorithm, altering the entropy, or directly extracting the features of the S-NSs, the proposed CEEMDAN-Single-Slopen approach had the highest recognition rate of 90.5%.
(3) The proposed CEEMDAN-Dual-Slopen approach further improved the classification performance of the CEEMDAN-Single-Slopen approach with a 7.1% improvement in recognition rate, and was better than other ship signal-based and IMF-based approaches under the dual-feature condition.
(4) Slopen was shown to be a good approach for extracting S-NSs features by analyzing the complexity of S-NSs. In the future, we will improve the slope entropy by combining the concepts of multi-scale and hierarchy to further improve the performance of the feature extraction approach.

## Figures and Tables

**Table 1:** The average recognition results of each IMF for three feature extraction approaches.

Approach	Average Recognition Rate (%)					
	IMF1	IMF2	IMF3	IMF4	IMF5	IMF6
EMD-Single-Slopen	60.3	59.8	64	58.1	43	29.6
EEMD-Single-Slopen	50	48.6	50.8	41.1	42.5	25
CEEMDAN-Single-Slopen	47.3	51.3	90.5	68.3	48.5	37.5

**Table 2:** The highest average recognition results of the dual-IMF optimized feature extraction approaches.

Method	Choose the IMFs	Average Recognition Rate (%)
EMD-Dual-Slopen	IMF3, IMF4	85.5
EEMD-Dual-Slopen	IMF1, IMF5	68.3
CEEMDAN-Dual-Slopen	IMF3, IMF5	97.6

**Table 3:** The average recognition results of every IMF of the four types of entropies.

Entropy	Average Recognition Rate (%)					
	IMF1	IMF2	IMF3	IMF4	IMF5	IMF6
Slopen	47.3	51.3	90.5	68.3	48.5	37.5
DE	70.3	89.5	66.5	47	55	51
RDE	72	85.3	64.6	49.6	52.1	50.1
FDE	76	90.3	52.1	52.8	58.8	51.1

**Table 4:** The highest average recognition rates of the dual-IMFs of the four types of entropies.

Entropy	Choose the IMFs	Average Recognition Rate (%)
Slopen	IMF3, IMF5	97.6
DE	IMF2, IMF6	95.6
RDE	IMF1, IMF2	92.5
FDE	IMF2, IMF5	94.8

**Table 5:** The average recognition rates of the proposed CEEMDAN-Single-Slopen approach and the four single-feature extraction approaches.

Subject	Feature	Average Recognition Rate (%)
IMFs of S-NSs	Slopen(3)	90.5
S-NSs	Slopen	64.6
S-NSs	DE	74.3
S-NSs	RDE	76.1
S-NSs	FDE	78.0

**Table 6:** The average recognition rates of the proposed CEEMDAN-Dual-Slopen approach and the dual-feature extraction approaches.

Subject	Features	Average Recognition Rate (%)
IMFs	Slopen(3) and Slopen(5)	97.6
Signals	Slopen and DE	95.6
Signals	Slopen and RDE	96.3
Signals	Slopen and FDE	95.3
Signals	DE and RDE	79.5
Signals	DE and FDE	94.5
Signals	RDE and FDE	92.0

## Data Availability

The data used to support the findings of this study are available from the corresponding author upon request.
